# Supplementary figures and images for: Ubiquitination of Ebola virus VP35 at lysine 309 regulates viral transcription and assembly
Source: PLoS Pathog. 2022 May 9;18(5):e1010532. doi: 10.1371/journal.ppat.1010532 (PMC9119628; doi:10.1371/journal.ppat.1010532)

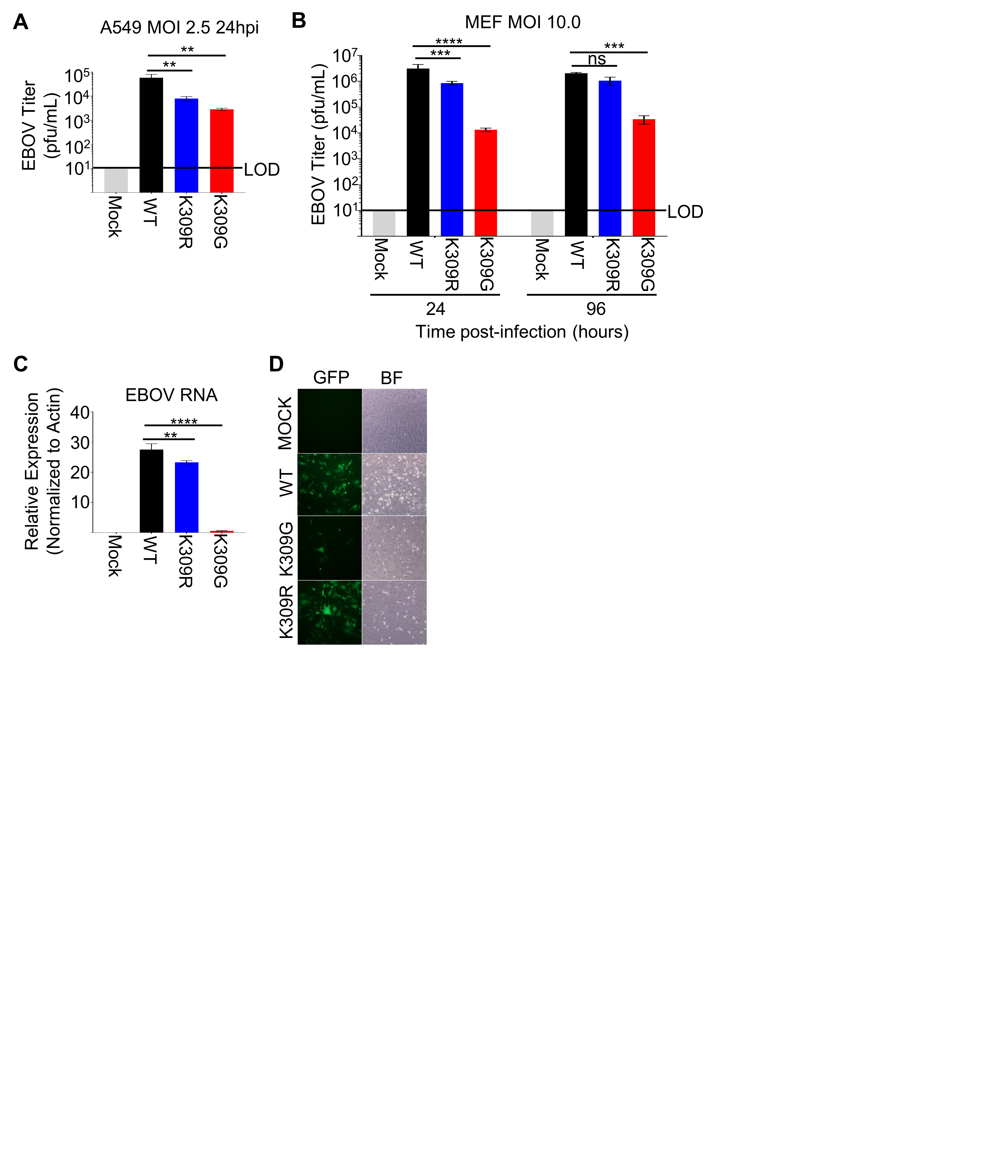

Supplement: S1 Fig — (A) A549 cells were infected with rEBOV-eGFP-VP35/wt, -K309R, or -K309G at an MOI of 2.5 PFU/cell for 24 hours. Limit of detection (LOD), 10 pfu/mL, is indicated. (B-D) Murine embryonic fibroblasts (MEFs) were infected with rEBOV-eGFP-VP35/wt, -K309R, or -K309G at an MOI of 10.0 PFU/cell. The titers are from 24 and 96 hpi, LOD 10pfu/mL (B), and EBOV RNA (C) and fluorescence microscopy images (D) are from 96 hpi. The titrations (A and B) and qRT-PCR (C) were done in triplicate. Analysis was done using a one-way ANOVA with Tukey’s post-test for comparison between groups. P-value: **< 0.01, ***<0.001, ****<0.0001; ns, not significant (p>0.05). (TIF) [file ppat.1010532.s001.TIF]

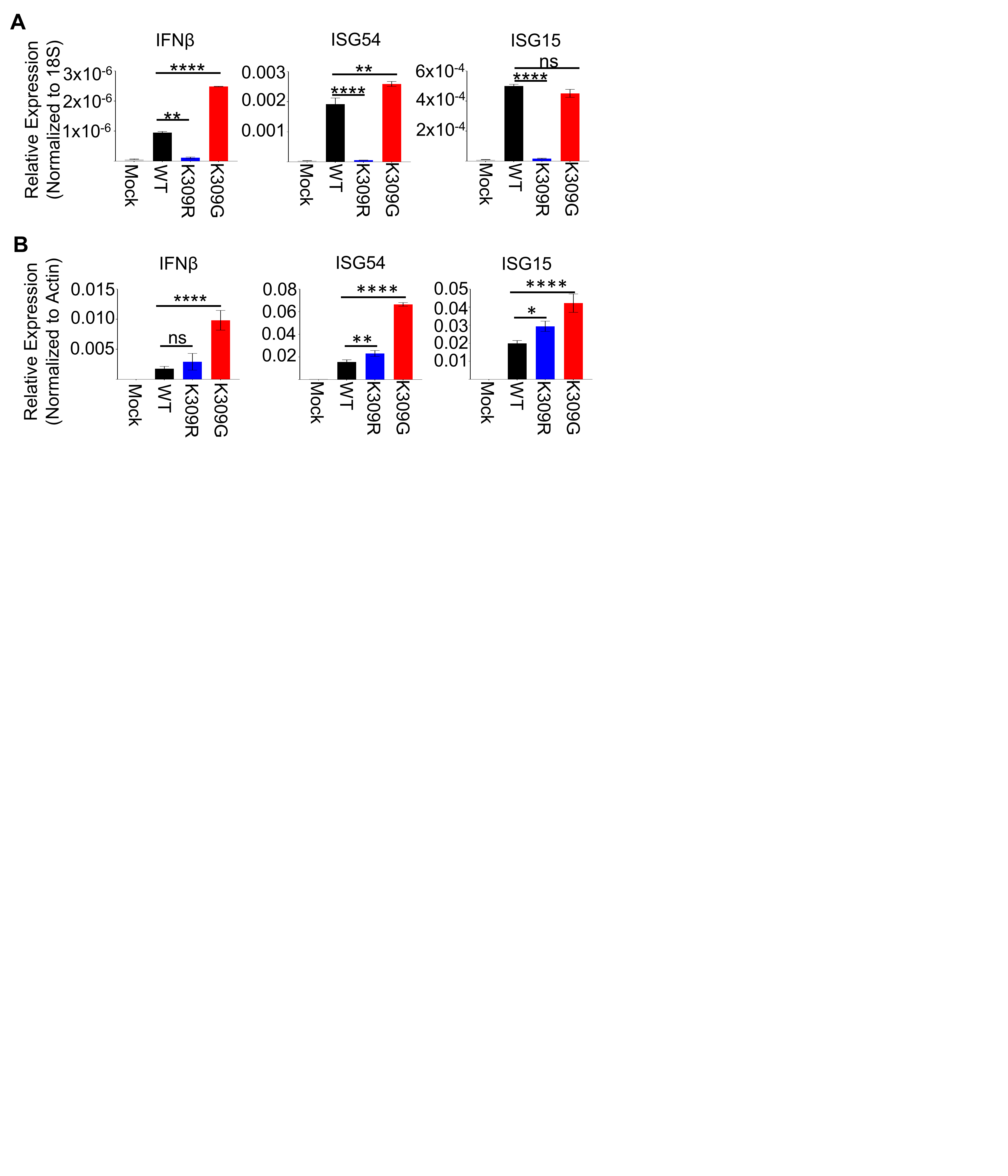

Supplement: S2 Fig — (A) A549 cells were infected with rEBOV-eGFP-VP35/wt, -K309R, or -K309G at an MOI of 2.5 PFU/cell for 24 hours and RNA was collected for qRT-PCR. (B) Murine embryonic fibroblasts (MEFs) were infected with rEBOV-eGFP-VP35/wt, -K309R, or -K309G at an MOI of 10.0 PFU/cell for 48 hours and RNA was collected for qRT-PCR. The Ifnb and interferon stimulated genes (ISGs) RNA cycle threshold value was normalized to the 18S value. Analysis was done using a one-way ANOVA with Tukey’s post-test for comparison between groups. P-value: *<0.05, **<0.01, ****<0.0001; ns, not significant (p>0.05). (TIF) [file ppat.1010532.s002.TIF]

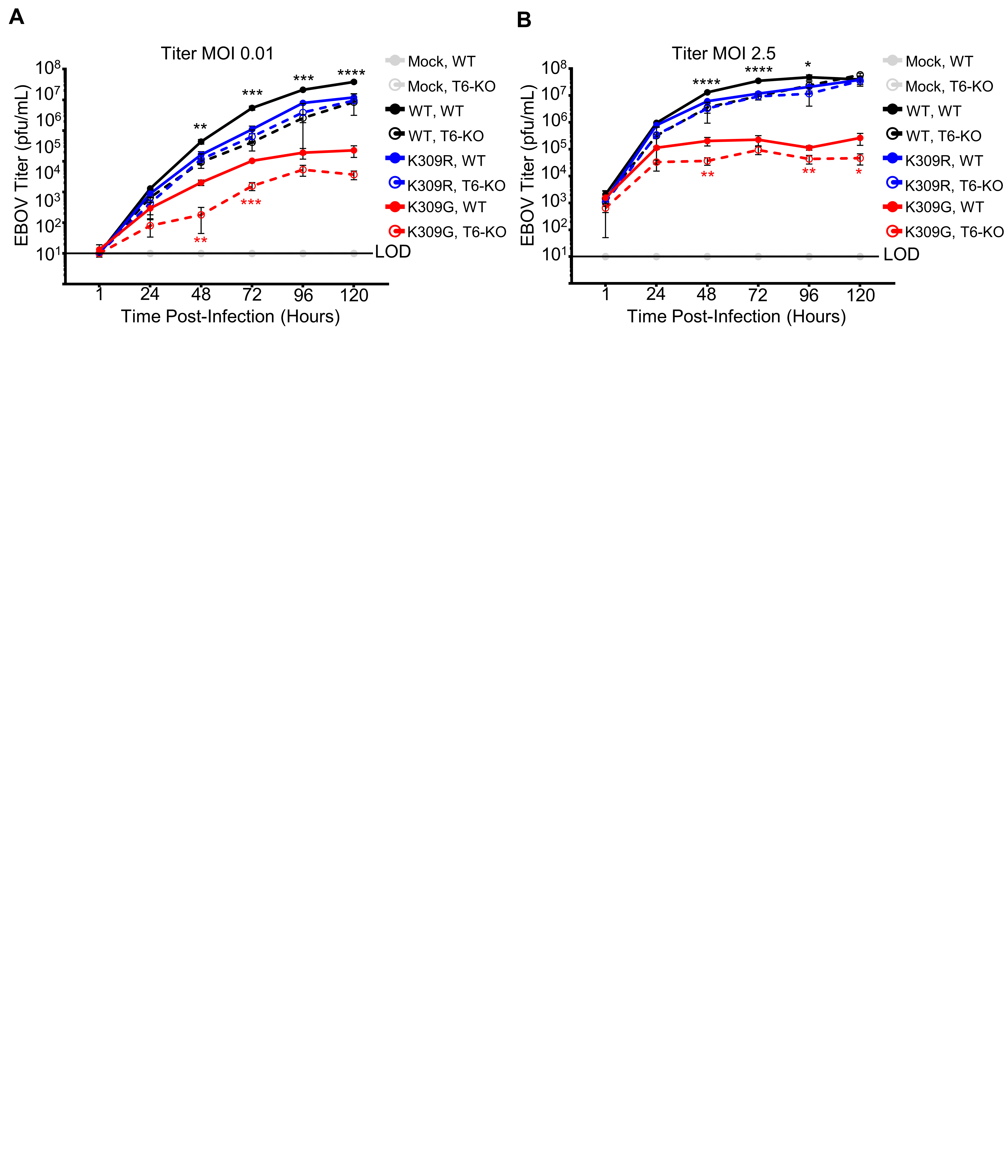

Supplement: S3 Fig — Wild-type (solid lines) and TRIM6 knockout (T6-KO) (dashed lines) A549 cells were infected with rEBOV-eGFP-VP35/wt, -K309R, or -K309G at multiplicity of infection (MOI) of 0.01 (A) and 2.5 (B) PFU/cell or mock treated (grey). The limit of detection (LOD) is 10 PFU/mL. The titrations were done in triplicate. The data analysis was done using a two-way ANOVA with Bonferroni’s or Tukey’s post-test for comparison between groups, respectively. P-value: *<0.05, ***<0.001, ****<0.0001. Black, red and blue stars represent wt, K309G and K309R comparison, respectively, between wt and T6-KO cells. Non-significant differences are not indicated to limit crowding on the graph. (TIF) [file ppat.1010532.s003.TIF]

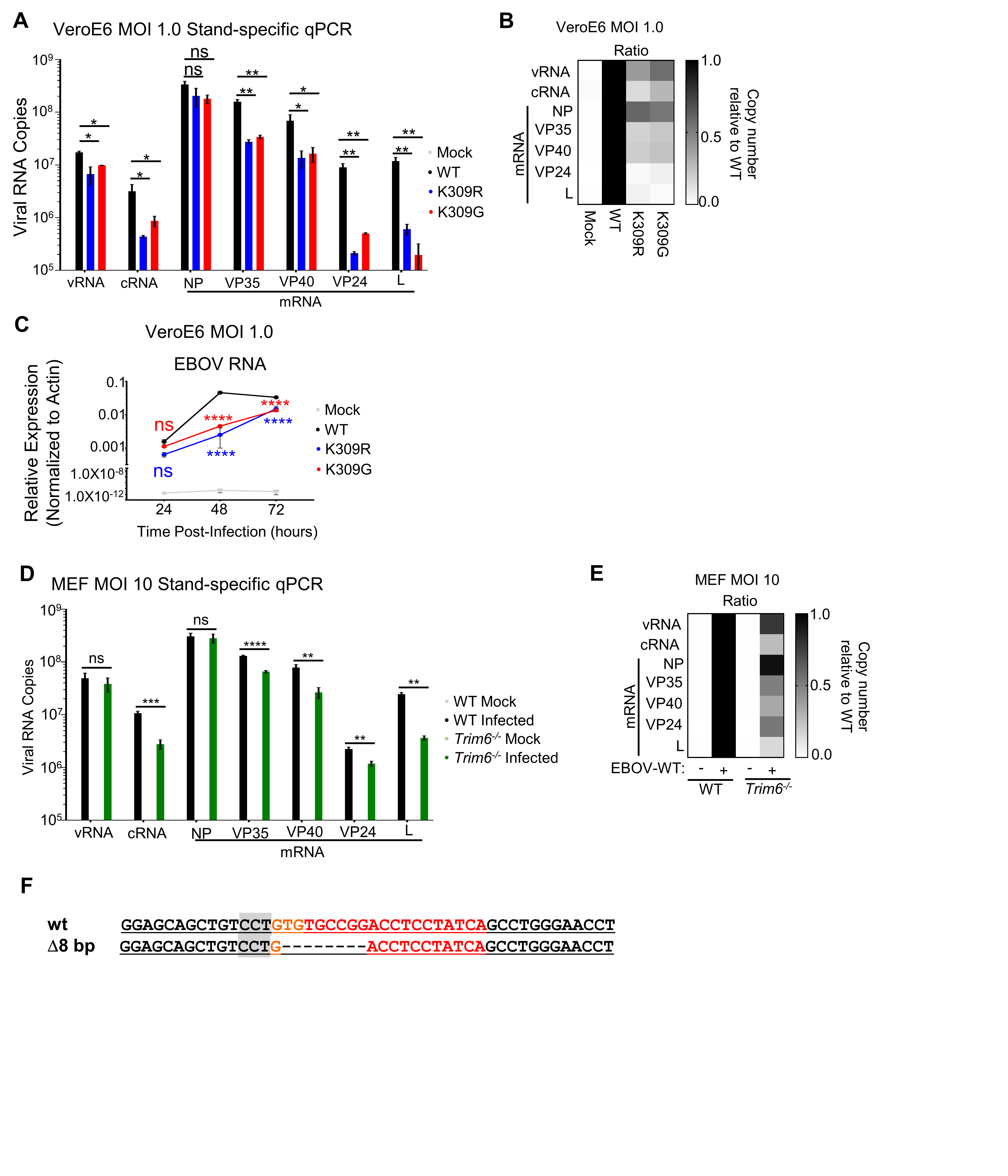

Supplement: S4 Fig — (A) VeroE6 cells were infected with rEBOV-eGFP-VP35/wt (black), -K309R (blue), or -K309G (red) at an MOI of 1.0 PFU/cell for 72 hours and RNA was collected for strand-specific qRT-PCR (two biological replicates run in triplicate). (B) Heat map representing the ratio of copy number relative to wt for each viral RNA species corresponding to the data presented in panel A. (C) Standard qPCR for viral RNA of VeroE6 cells at 24, 48, and 72 hpi (corresponding to samples used in Fig 3C, 3D and panel A). The EBOV RNA signal was normalized to the 18S cycle threshold value. (D) WT (black) or Trim6-/- (green) murine embryonic fibroblasts (MEFs) were infected with rEBOV-eGFP-VP35/wt at an MOI of 10.0 PFU/cell for 96 hours and RNA was collected for strand-specific qRT-PCR (triplicates). (E) Heat map representing the ratio of copy number relative to wt for each viral RNA species corresponding to the data presented in panel D. (F) The 8bp deletion (Δ8bp) in TRIM6 sequence of the TRIM6-KO mice used for MEF and bone marrow-derived dendritic cell generation. The highlighted sequence corresponds to the PAM, the sequence in red is the sgRNA target sequence, and the dashes represent the deleted nucleotides. The data analysis was done using a two-way ANOVA with Bonferroni’s post-test for comparison between groups (C), one-way ANOVA with Tukey’s post-test for comparison between groups (A) or a student’s t-test (D). P-value: * <0.05, **<0.01, ***<0.001, ****<0.0001; ns, not significant (p>0.05). (TIF) [file ppat.1010532.s004.TIF]

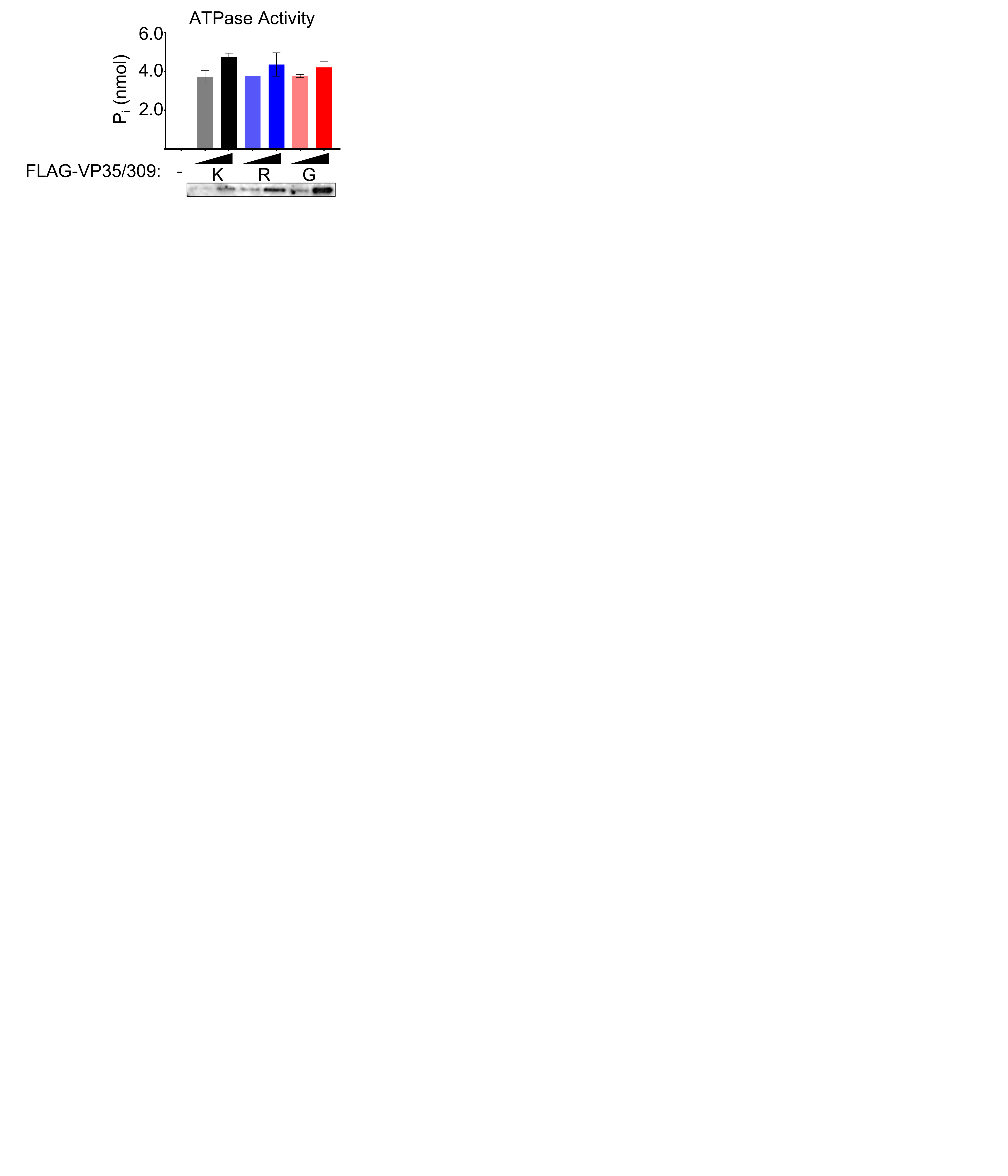

Supplement: S5 Fig — FLAG-purified VP35 (wt, K309R, or K309G) was used in an ATPase activity assay. The concentration of free phosphate (Pi) was determined using a standard curve with the BIOMOL Green phosphate standard. A fraction of the completed reaction was boiled in 4X Laemmli sample buffer to compare the amount of VP35 added. The assay was completed in biological triplicate. The data analysis was done using a one-way ANOVA with Tukey’s post-test for comparison between groups. No significant differences were identified. (TIF) [file ppat.1010532.s005.TIF]

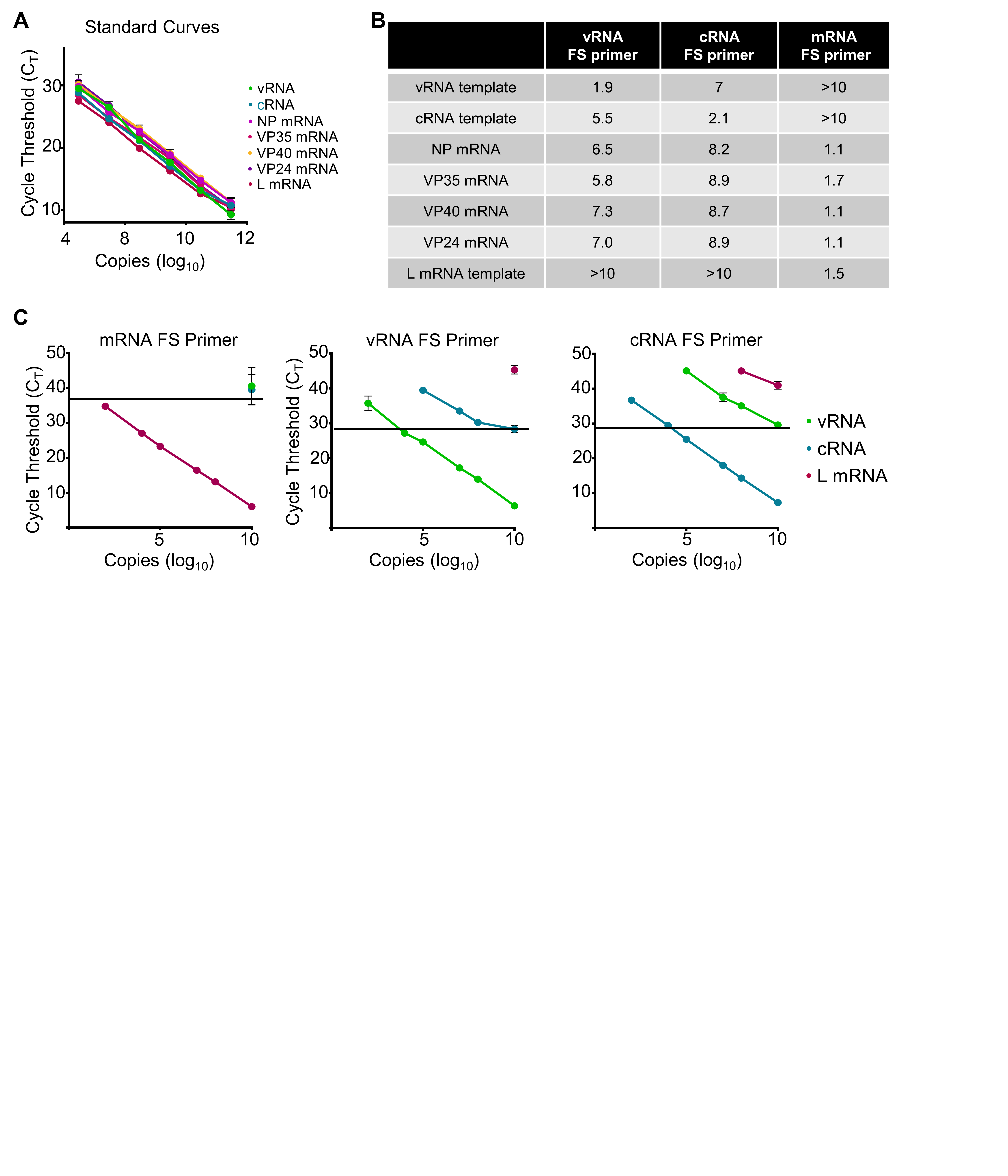

Supplement: S6 Fig — (A) Representative standard curves for each viral RNA species tested, genomic RNA (vRNA), anti-genomic RNA (cRNA), and NP, VP35, VP40, VP24, and L transcripts (mRNA) run along with each strand-specific qPCR run. (B) Table of the limit of detection (log10 copies) for each first-strand cDNA primer-in vitro transcript pair. (C) Graphical representation of how cycle threshold cut off values were determined for each first-strand primer/qPCR pair. (TIF) [file ppat.1010532.s006.TIF]
