# Supplementary material for: Ubiquitination of Ebola virus VP35 at lysine 309 regulates viral transcription and assembly
Source: PLoS Pathog. 2022 May 9;18(5):e1010532. doi: 10.1371/journal.ppat.1010532 (PMC9119628; doi:10.1371/journal.ppat.1010532)
Supplement: S1 Table — A list of all used primer sequences. Sequences are listed 5’-to-3’. (DOCX) [file ppat.1010532.s007.docx]

| **Table S1. Primer Sequences** | | | |  |
| --- | --- | --- | --- | --- |
| Standard qPCR | EBOV F | TTTTCAATCCTCAACCGTAAGGC |  |  |
|  | EBOV R | CAGTCCGGTCCCAGAATGTG |  |  |
|  | Human IFNβ F | TCTGGCACAACAGGTAGTAGGC |  |  |
|  | Human Ifnβ R | GAGAAGCACAACAGGAGAGCAA |  |  |
|  | Human ISG54 F | ATGTGCAACCCTACTGGCCTAT |  |  |
|  | Human ISG54 R | TGAGAGTCGGCCCAGTGATA |  |  |
|  | Human Mx1 F | GGCTGTTTACCAGACTCCGACA |  |  |
|  | Human Mx1 R | CACAAAGCCTGGCAGCTCTCTA |  |  |
|  | Human ISG15 F | TCCTGGTGAGGAATAACAAGGG |  |  |
|  | Human ISG15 R | GTCAGCCAGAACAGGTCGTC |  |  |
|  | Human Ddx58 F | GGCATGTTACACAGCTGACG |  |  |
|  | Human Ddx58 R | TGCAATATCCTCCACCACAA |  |  |
|  | 18S F | GTAACCCGTTGAACCCCATT |  |  |
|  | 18S R | CCATCCAATCGGTAGTAGCG |  |  |
|  | Mouse IFNβ F | CAGTCCAAGAAAGGACGAAC |  |  |
|  | Mouse IFNβ R | GGCAGTGTAACTCTTCTGCAT |  |  |
|  | Mouse ISG54 F | CTGAGAGGGGAGTGGACTCTG |  |  |
|  | Mouse ISG54 R | GCACCTGCTTCATCCAAAGAT |  |  |
|  | Mouse ISG15 F | GCAAGCAGCCAGAAGCAGACTCC |  |  |
|  | Mouse ISG15 R | CGGACACCAGGAAATCGTTACCCC |  |  |
|  | Mouse Actin F | CGGTTCCGATGCCCTGAGGCTCTT |  |  |
|  | Mouse Actin R | CGTCACACTTCATGATGGAATTGA |  |  |
| In vitro Transcript  Primers | vRNA F | CATAGTATCCTGATACTTGCAAAGGT |  |  |
|  | vRNA T7 R | ggatcctaatacgactcactatagggTGGACACACAAAAAAGAAGAAATAGA |  |  |
|  | cRNA T7 F | ggatcctaatacgactcactatagggCATAGTATCCTGATACTTGCAAAGGT |  |  |
|  | cRNA R | TGGACACACAAAAAAGAAGAAATAGA |  |  |
|  | mRNA R | TTTTTTTTTTTTTTTTATAAT |  |  |
|  | L mRNA T7 F | ggatcctaatacgactcactatagggCTCCCGCAAGACGAGCAACAAGATCAGGACCACACT |  |  |
| In vitro Transcript  Primers | NP mRNA T7 F | GGATCCTAATACGACTCACTATAGGG GATGAAGGATGAGCCTGTAG |  |  |
|  | VP35 mRNA T7 F | GGATCCTAATACGACTCACTATAGGG TCCGCTCTCGAGGTGACA |  |  |
|  | VP40 mRNA T7 F | GGATCCTAATACGACTCACTATAGGG TCACCATGGTAATCACACA |  |  |
|  | VP24 mRNA T7 F | GGATCCTAATACGACTCACTATAGGG ATGAGTCCACACTGAAAG |  |  |
| First-Strand cDNA Synthesis Primers | FS vRNA | GGCCGTCATGGTGGCGAATagctttaacgaaaggtctgggctc |  |  |
|  | FS cRNA | GCTAGCTTCAGCTAGGCATCacacaaagattaaggctatcaccg |  |  |
|  | FS mRNA | CCAGATCGTTCGAGTCGTTTTTTTTTTTTTTTTCTTAAT |  |  |
| Strand-Specific qPCR Primers | vRNA qPCR tag F | GGCCGTCATGGTGGCGAAT |  |  |
|  | vRNA qPCR R | acacaaagattaaggctatcaccg |  |  |
|  | cRNA qPCR F | agctttaacgaaaggtctgggctc |  |  |
|  | cRNA qPCR tag R | GCTAGCTTCAGCTAGGCATC |  |  |
|  | mRNA qPCR tag R | CCAGATCGTTCGAGTCGT |  |  |
|  | L mRNA qPCR F | ATTGCAATTGTGAAGAACGTTTC |  |  |
|  | NP mRNA qPCR F | GATGAAGGATGAGCCTGTAG |  |  |
|  | NP mRNA qPCR R | CCATGGTGGATATTCCTC |  |  |
|  | VP35 mRNA qPCR F | TCCGCTCTCGAGGTGACA |  |  |
|  | VP35 mRNA qPCR R | CAACCTCGATCAATCTTG |  |  |
|  | VP40 mRNA qPCR F | TCACCATGGTAATCACACA |  |  |
|  | VP40 mRNA qPCR R | TTCTCAATCACAGCTGGAA |  |  |
|  | VP24 mRNA qPCR F | ATGAGTCCACACTGAAAG |  |  |
|  | VP24 mRNA qPCR R | GATAGCAAGAGAGCTATT |  |  |
| Cloning Primers | pCAGGS VP35 NheI R | TATAATGCTAGCTCAAATTTTGAGTCCAAGTGTTTTACCATCT |  |  |
|  | pCAGGS VP35 K309R XhoI F | ATTTCTCGAGGTGACATTCCCCGAGCTTGCCAGAGAAGCTTGCGTCCAGTCCCA |  |  |
|  | pCAGGS VP35 K309G XhoI R | ATTATACTCGAGGTGACATTCCCCGAGCTTGCCAGGGAAGCTTGCGTCCAGT |  |  |
|  | pQE-VP35 KpnI F | ATTATAGGTACCATGACAACTAGAACAAAGGGCA |  |  |
|  | pQE-VP35 NotI R | TATAATGCGGCCGCAATTTTGAGTCCAAGTGTTTTACCA |  |  |
| Cloning Primers | VP35 N-C Overlap K216,222R F | CTAACTGAGGAAAATTTTGGGAGACCTGACATTTCGGCAAGGGATTTGAG |  |  |
|  |  | AAACATTATG |  |  |
|  | VP35 N-C Overlap K216,222R R | CATAATGTTTCTCAAATCCCTTGCCGAAATGTCAGGTCTCCCAAAATTTT |  |  |
|  |  | CCTCAGTTAG |  |  |
|  | VP35 K63,67 F | GCATCCCAAATGCAACAAACGAAGCCAAACCCGAAGACGCGCAACAGTCA |  |  |
|  |  | AACCCAAAC |  |  |
|  | VP35 K63,67 R | GTTTGGGTTTGACTGTTGCGCGTCTTCGGGTTTGGCTTCGTTTGTTGCAT |  |  |
|  |  | TTGGGATGC |  |  |
|  | VP35 K319, 334,339R NheI R | AAGCTAGCTCAAATTCTGAGTCCAAGTGTTCTACCATCTTGAAGCTGAAAAACACATACCCAA CCTCGATCAATCCTGGGCGATG |  |  |
|  | VP35 K119.126.141R AgeI R | AATAACCGGTTGTCATCACCAGAAGATCATATCTTGCAACCATCTCAGCACAAACCCTGTTC AATGAGGAGATTGTTCTTGCCATATCATAAACTGGCCTTAGACCATT |  |  |
|  | VP35 K6R NotI F | TATTGCGGCCGCAATGACAACTAGAACAAGGGGCAGGGGCCATACTGCGG |  |  |
|  | VP35 K282R XhoI R | TATTCTCGAGAGCGGATGTGGATGACAGGTGGAGCAGCATCTTGGAAGATTGGAACTCTTCTTGTA ATTTGAATTAGGGCACA |  |  |
|  | VP35 K222,248,252R F | GCAAGGGATTTGAGAAACATTATGTATGATCACTTGCCTGGTTTTGGAACTGCTTTCCACCAATTAGTACAAGTGATTTGTAGATTGGGAAGAGATAGC |  |  |
|  | VP35 K222,248,252R R | GCTATCTCTTCCCAATCTACAAATCACTTGTACTAATTGGTGGAAAGCAGTTCCAAAACCAGGCAAGTGATCATACATAATGTTTCTCAAATCCCTTGC |  |  |
|  | VP35 K184R AgeI-F | ACCGGTCGGGCAACAGCAACCGCTGCGGCAACTGAGGCTTATTGGGCCGAACATGGTCAACCACCACCTGGACCATCACTTTATGAAGAAAGTGCGATTCGGGGTAGGATTGA |  |  |
| Cloning Primers | TRIM6 F pCMV SgfI | ATTATAGCGATCGCCATGACTTCACCAGTACTGGTGGA |  |  |
|  | TRIM6 R pCMV Mlu | TATAATACGCGTAGAGCTTGGACGACGCAGGGTCAT |  |  |
| Sequencing Primers | pCAGGS F | GCCTCTGCTAACCATGTTCAT |  |  |
|  | pCAGGS R | CCAACACACTATTGCAATGAAA |  |  |
|  | pQE F | GGTTATTGTGCTGTCTCATC |  |  |
|  | pQE R | TCGATCTCAGTGGTATTTGTG |  |  |
| Recombinant Virus Cloning | VP35 K309R F | AGCTTGCCAGCGGAGCTTGCGTC |  |  |
|  | VP35 K309G F | AGCTTGCCAGGGCAGCTTGCGTC |  |  |
|  | VP35 R | CGGGGAATGTCACCTCGA |  |  |
| Knockout Mouse Generation | sgRNA *Trim*6 exon 2 | TGATAGGAGGTCCGGCACAC |  |  |
| Knockout Mouse Screening PCR | *Trim6* F | GCTGGGAAGGTAGGAGACGA |  |  |
|  | *Trim6* R | GAGATCGTTCACAAAGCCAGC |  |  |
