# Supplementary material for: Ubiquitination of Ebola virus VP35 at lysine 309 regulates viral transcription and assembly
Source: PLoS Pathog. 2022 May 9;18(5):e1010532. doi: 10.1371/journal.ppat.1010532 (PMC9119628; doi:10.1371/journal.ppat.1010532)
Supplement: Attachment — Submitted filename: Response to Reviewers PPATHOGENS-D-21-02278.docx [file ppat.1010532.s008.docx]

18 March 2022

PPATHOGENS-D-21-02278

**Point-by-point responses (in red) to the reviewer’s comments (in black)**

*For brevity, only the reviewer’s comments/critiques (without the summaries) are shown below. The full reports (with summaries) are included at the end of the document.*

**REVIEWER 1**

**Part I - Summary**

**Please use this section to discuss strengths/weaknesses of study, novelty/significance, general execution and scholarship.**

Reviewer #1: In this manuscript, the authors continue their research on the role of Ebola virus VP35 ubiquitination for viral replication. By intensively investigations of two VP35 mutants, carrying substitutions at amino acids 309 to arginine or glycine residue, K309R or K309G, the authors could show by applying reporter gene assays, interaction studies and recombinant viruses, that ubiquitination of VP35 K309 is essential for interaction with the viral polymerase L in order to initiate efficient viral RNA synthesis. In contrast, lack of VP35 ubiquitination leads to stronger interaction with NP, allowing nucleocapsid assembly and packaging into progeny virions. All together, these results suggest ubiquitination of Ebola virus VP35 plays a key role in switching the two different functions of VP35 in transcription vs nucleocapsid assembly.

The manuscript is very well written and provides new evidence for the mechanistic role of VP35 ubiquitination during viral infection by modulating the two distinct function of VP35 in transcription and nucleocapsid assembly through ubiquitination of VP35 K309. The experiments are solidly executed and are very intensively controlled.

There are only minor issues to be addressed

We first would like to thank the reviewer for taking the time to review our manuscript. We also thank the reviewer for their overall positive and encouraging comments. We thank the reviewer for the constructive criticism which we believe has strengthened the quality of our manuscript.

**Major Issues**

Reviewer #1: There are only minor issues to be addressed

**Minor Issues**

Reviewer #1:

1. Line 83: the abbreviation for antigenomic RNA, agRNA, is unusual. Conventionally, the terms and abbreviations for vRNA (genomic RNA) and cRNA (copy RNA, antigenome) are more widely used.

Reply: We thank the reviewer for this comment. As recommended, we have changed all notations of anti-genomic RNA as cRNA and genomic RNA as vRNA.

2. Line 175: To this reviewer, usage of A549 cells, a lung cell line, for Ebola virus infection seems unusual. There are more widely used IFN competent cells like Huh7 cells, a hepatoma cell line, liver cells are also infected during an authentic viral infections. Could the authors please comment on their rational to use the A549 cells?

Reply: We appreciate the reviewer’s concern. We elected to use A549 cells because we had previously generated TRIM6 CRISPPR knockout in this A549 cells and had previously characterized EBOV replication kinetics thoroughly in these cells. We believe that the effects are not highly dependent on cell types, since we observe similar results using primary murine fibroblasts and bone marrow-derived dendritic cells (BMDCs) from *Trim6^-/-^* mice. We do agree with the reviewer that some effects on EBOV replication could vary depending on the cell types and we cannot rule it out, but we believe that the effects are robust and consistent across species (human vs mice). We also believe that using primary BMDCs makes the results significant and relevant. We have addressed this matter in the discussion section (lines 451-455; 457-459).

3. Line 190 and line 193 Typo Ifnb. Must be IFNß. Isg must be ISG

Reply: We thank the reviewer for pointing this out. We have corrected the typos.

4. Fig. 4A. To me, it is not clear why the authors also assed reporter gene activity at lower doses of VP35 and mutants. The ratio between VP35 and NP in the minigenome assay setting (Hoenen et al., 2004, Wenigenrath et al., 2010) is very critical for efficient transcriptional activity. Altering this ratio by just transfecting a quarter of the usual dose of VP35 will of course affect transcriptional activity. However, this must not have anything to do with the VP35 Ub mutations at all.

Reply: We thank the reviewer for this thoughtful comment. In our experience, we have seen that the effects of VP35 in the minigenome are highly dependent on concentration and as pointed out by the reviewer on the ration of VP35:NP. We used the lower dose to observe the dose response, but also to mimic the NP:VP35 ratio earlier during infection. We were curious as whether ubiquitination at this site had an effect at this higher NP:VP35 ratio. Similar to what is seen with viral RNA in Figures 2B and 2E, we don’t see a difference in total viral RNA early during infection (24 hpi) when the NP:VP35 ratio is higher. We have included a sentence on this in the paper to make the rationale clearer (line 247-248).

5. Fig. 6C. Expression control of L is missing

Reply: We thank the reviewer for catching this. The lysates from figure 6C were re-run and the resulting HA and tubulin blots are now included.

6. Fig. 6F. A quantification of Western Blots is missing. While the authors state that interaction of mutants with VP30 is not impaired (line 346), the blot shows another picture. Also with respect to Fig 7A, where K309G infection shows reduced VP30 incorporation, it is highly recommended to include here a statistical analysis of more than 1 Blot to interpret these results more precisely.

Reply: We thank the reviewer for their comment, and we have included the quantifications of these Figures from three replicate western blots (new quantification for Figures 8A and 8B). Clearer western blot image panels have also been substituted. As the reviewer suggested, quantification shows no significant differences for VP30 incorporations into K309G mutant virus particles (Figure 8B).

7. Fig. 8. It is highly recommended to replace the reddish, pinkish colours with different types to improve clarity of the model. Also the particles that are “produced” are really hard to recognize instinctively.

Reply: We thank the reviewer for suggesting a way to improve the interpretability of our summary Figure (now Figure 9). We have changed the colors of the EBOV proteins to make them more distinct, and we added a black outline to the shapes to enhance contrast. The EBOV particles have also been removed and left only the text to improve readability.

**REVIEWER 2**

**Part I – Summary**

Reviewer #2: This manuscript by van Tol et al., explores the role of Ebola VP35 ubiquitination. This work builds on a previous observation that Ebola VP35 is ubiquitinated on a specific basic residue, K309. The authors extend this initial observation by generating recombinant viruses with mutations at K309 (K209G and K309R) in an attempt to define the molecular mechanism. Much of the data in the manuscript support the original observation from the same group, Bharaj et al. 2017, with the major contribution, albeit not entirely surprising is the fact that mutations within the C-terminal RNA binding interferon inhibitory domain (RBD/IID) does not impact its interferon inhibitory function. This is consistent with multiple prior reports which suggested that K309 plays a minor role in the central basic patch region. Authors also generated recombinant viruses and this was important and has the potential to impact our knowledge. While the role of posttranslational modifications, including ubiquitination are likely to impact Ebola replication, the data in the manuscript overall is not rigorous. Often the error bars/statistical considerations are missing and the data/replicates are missing. Therefore, while the manuscript presents and important idea, the significance and the impact of the data lacks an ability to support the hypothesis. For example data in Figure 3 shows a log difference in panel A, but a black and white difference in panel C at MOI of 5 and 48 hours time. Panel C is using a normalization to actin. Using multiple Y-axis, the authors are either trying to convince themselves and the reader or enhance a modest effect. Unclear which of this is true. Finally, data in Figure 6 is intriguing. If the ubiquitination was truly the reason for why VP35 does not bind to Ebola viral L, then using WT VP35 and WT L1-505, one should see a disruption in TRIM6 KO cells. That data is missing. Again, in multiple instances, the authors do not provide rigorous data to support their ideas. For these reasons, there is promise, but does not deliver with respect to biology.

Reply: We first would like to thank the reviewer for taking the time to review our manuscript. We are sorry that the reviewer found our manuscript not rigorous, and we have made a genuine attempt to address the reviewer’s criticisms. We believe that some information might have been lost to the reviewer or might have not been clear, and we have made an effort to clarify and address all concerns. We believe the reviewers comments have helped us improve the clarity and quality of our manuscript, and we thank the reviewer for their criticism which we believe has strengthened the quality of our manuscript.

Specifically, we would like to point out that all but one Figure (Figure 2K) with quantification data included error bars in the original submission, and that has been addressed now (see below). As mentioned in the methods section of the paper, the information on the replication of each experiment was included in the figure legends. We agree with the reviewer’s comments that performing binding experiments in wt and TRIM6 knockout cells would improve the strength of our conclusions, and we included three new experiments to address this concern. We think that our data tests our hypothesis from multiple experimental approaches that support our conclusions, and demonstrates the rigor by using knockout cell lines, primary dendritic cells from knockout mice, and recombinant infectious mutant viruses that have never been generated before. Please see below specific answers to the reviewer’s comments.

**Major Issues**

Reviewer #2:

Figure 3 shows a log difference in panel A, but a black and white difference in panel C at MOI of 5 and 48 hours time. Panel C is using a normalization to actin. Using multiple Y-axis, the authors are either trying to convince themselves and the reader or enhance a modest effect. Unclear which of this is true.

Reply: We are sorry that the normalizations were unclear. The data presented in Figure 3A was titration data from wild-type (WT) and TRIM6 (T6) knockout (KO) A549 cells infected at a multiplicity of infection (MOI) 5.0 for 48 hours, while Figure 3C was for qPCR data from primary murine bone marrow derived dendritic cells (BMDCs) infected at MOI 0.5 for 24 hours. These data are not directly comparable as they are from different cell types, infection conditions, and readouts. The data in panel 3C was normalized to actin since this was qPCR (to measure viral RNA in infected cells), and we had not collected supernatants for titrations for the corresponding samples. To specifically address the reviewer’s issue, we repeated the experiment by completing a full replication kinetics (new Figures 3A-D and S3A and S3B) in WT and T6-KO cells with the WT or VP35/K309 mutant viruses at both low (0.01) and high (2.5) MOIs to replicate the conditions used in Figure 2. Our new results are consistent with the original Figure 3A showing that WT virus is attenuated in T6-KO cells to a similar degree as the K309R mutant in either WT or T6-KO cells (new Figure 3). To address the issue of the normalization, we have now measured the absolute EBOV RNA copy number in the BMDC samples to avoid the normalization to actin (new Figure 3E). The EBOV genome copy number shows that the results are consistent with our original Figure 3C. Since early during infection, as seen in Figures 2B and 2D, we did not expect to observe large differences in viral RNA at 24 hours in BMDCs. At 24hpi, minimal virus is produced, and we did not investigate additional time points initially. To address potential differences in viral replication, we extended the duration of infection in BMDCs to 96 hours following infection with either the WT or K309R virus at MOI 0.5 (new Figure 3F). As with infection in A549 cells, we observe attenuation of the WT virus *in* *Trim6^-/-^* BMDCs and K309R virus in WT and *Trim6^-/-^* BMDCs compared to WT virus in WT cells (new Figure 3F). These data support that TRIM6-facilitated ubiquitination of EBOV VP35 K309 supports virus replication.

Data in Figure 6 is intriguing. If the ubiquitination was truly the reason for why VP35 does not bind to Ebola viral L, then using WT VP35 and WT L 1-505, one should see a disruption in TRIM6-KO cells. That data is missing.

Reply: This is an excellent suggestion. We thank the reviewer for this comment, which we believe enhances the relevance of our findings. We agree with the reviewer that evaluating VP35 binding with L and NP in wild-type or TRIM6 knockout cells provides stronger evidence for TRIM6-mediated ubiquitination in facilitating the observed phenotypes. We have added two new experiments to support that TRIM6-mediated VP35 ubiquitination facilitates optimal interaction with EBOV L. In the first experiment, we over-expressed VP35 and HA-L_1-505_ in WT or T6-KO cells and immunoprecipitated HA-L_1-505_ (new Figure 6D). We also see endogenous TRIM6 immunoprecipitating with HA-L_1-505_, which supports that VP35, L, and TRIM6 may be forming a complex (new Figure 6D). As expected, HA-L_1-505_ binding to VP35 was impaired in T6-KO cells. Further, we rescued T6-KO cells with WT or catalytic mutant (C15A) TRIM6 to examine the importance of TRIM6’s ubiquitin ligase activity in facilitating binding to EBOV L (new Figure 6E). We observe that co-expression with WT, but not C15A, TRIM6 enhances VP35’s interaction with HA-L_1-505_ which further supports our hypothesis.

We also performed an analogous experiment to evaluate the dependence of TRIM6-mediated VP35 ubiquitination on impeding full binding to nucleoprotein (new Figure 7B). Consistent with our results in Figure 7A showing strong NP binding for the VP35/K309R mutant, we observed that VP35-HA-NP binding is stronger in T6-KO than in WT cells.

**Minor Issues**

Reviewer #2 Comments:

Statistics/repeats: In all the figure legends we have ensured that we included information on the number of replicates. We also double-checked all of the figure legends that describe the statistical tests applied, and we clarified that the comparisons with p-value >0.05 (this is specifically for the two-way ANOVAs) are not marked. We did not mark p-value >0.05 on two-way ANOVA data to keep from having too cluttered of figures. The only figure that did not have error bars in the original submission, Figure 2K, was repeated.

Use of acceptable Y-axis: We used the appropriate Y-axis for all Figures. We assume the reviewer is referring to the Figure 3A and -C discrepancy mentioned in their summary comment about having normalized to actin. Since Figure 3C was qPCR data from mouse cells with actin as the control gene, this was the appropriate label for the data presented. This original figure 3C was replaced with a genomic RNA copy number figure, which avoids the normalization with actin. The experiment shows a very similar result to the original figure.

Unusual nationalizations: We think the reviewer may be referring to “normalizations” regarding the comment on normalizing virus RNA to actin. We have now shown absolute copy numbers. We have made sure that all other normalizations have been clearly described in the methods results, and/or figure legends. The lines pertaining to the normalization information for each figure panel is listed below for convenience.

Figure 1B: Lines 942-945

Figure 1E: Lines 952-956

Figure 1F: Lines 958-962

Figure 1G: Lines 965-968

Figure 2K: Lines 983-987

Figure 6A: Lines 1056-1058

Figure 6B: Lines 1060-1062

Figure 6C: Lines 1064-1066

Figure 6D: Lines 1069-1071

Figure 6E: Lines 1074-1077

Figure 7A: Lines 1085-1087

Figure 7B: Lines 1089-1091

Figure 7E: Lines 1106-1108

Figure 7F: Lines 1110-1112

Figure 8A: Lines 1124-1126

Figure 8B: Lines 1130- 1131

Figure 8D: Lines 1133-1135

Figure 8E: Lines 1135-1138

Figure 8F: Lines 1139-1141

**REVIWER 3**

**Part I - Summary**

Reviewer #3: This is a thoroughly designed and executed study demonstrating the significance of ubiquitination of EBOV VP35 at lysine 309 in viral transcription and assembly. The authors generated recombinant K309R and K309G EBOV mutants to differentiate the role of ubiquitination and the basic property of the K309 residue in regulating virus infection. It appears that Lysine ubiquitination is not essential for VP35 to antagonize IFN-1 activity but was necessary for transcription and proper virus assembly. Several approaches have been used to drive the main conclusions. The data is also supported by rigorous statistical analysis.

The rescue of the mutant viruses and demonstration of their attenuating phenotype in vitro assays by itself is very promising for developing countermeasures. This is further enhanced by detailed molecular studies into the mechanism of action.

Reply: We would like to thank the reviewer for taking the time to review our manuscript and for their overall positive and encouraging comments. We thank the reviewer for the constructive criticism which we believe has strengthened the quality of our manuscript. We specifically thank the reviewer for their helpful suggestion on how to improve the experiment evaluating the influence of ubiquitination removal on VP35-NP binding (described below).

Reviewer #3: I have one concern that needs clarification:

Figure 6E. This is a very nice experiment however interpretation of the results didn’t seem right due to the following reasons: 1) It appears that OTU co-expression with VP35 or VP35-K309R was sufficient to enhance VP35-NP interaction. This appears to be the case at both low and high expression of VP35. However, the corresponding densitometry analysis didn’t correlate with the Western blot data. Based on Western blot alone, it appears that there is some ubiquitin-dependent components that was presumably destroyed by OTU that enhanced NP-VP35 interaction. This is in addition to the enhancement of NP-VP35 due to OTU mediated loss of ubiquitin modification at K309. 2) Can the authors demonstrate, as a control, the level of expression of the ubiquitinated-VP35 species in the lysates of mock, OTU and 2A transfected cells. This will also check if there are any additional residues on VP35 (other than K309) that are ubiquitinated (like in figure 1C). It is possible that these additional residues are influencing VP35-NP interaction.

Reply: We thank the reviewer for their insightful comment, and excellent suggestion. We appreciate their suggestion to demonstrate the level of ubiquitin associated with VP35 following OTU treatment. To address the first part of this comment, first, we would like to point out that these experiments are very challenging due to the transfection of multiple plasmids, and the overexpression of the OTU, which is very potent in deubiquitinating proteins. To ensure reproducibility, we had performed the experiments multiple times. The apparent discrepancy in the densitometry is due to unequal expression from the one representative experiment selected for the Figure.

To address both aspects of the reviewer’s comments, we repeated the experiment and added an additional VP35 mutant that had all sixteen of its lysine residues mutated to arginine (K-all-R) and added the lysates to beads with HA-NP, as before, or anti-VP35 antibody (new Figures 7C-F). Consistent with the original experiment, we observe treatment with WT OTU, but not the OTU 2A, enhances VP35 WT’s binding to HA-NP (new Figures 7D and 7F). We also find stronger binding of VP35 K309R than WT to HA-NP in the absence of OTU, and there is no difference in how well K309R or K-all-R bound to HA-NP in the OTU WT or -2A treated lysates. By pulling down for VP35 from the same lysates, we show that WT VP35 pulls down more ubiquitin than either the K309R or K-all-R mutants and that OTU WT effectively removes all ubiquitin (new Figures 7D and 7E). These results further support that the ubiquitination at VP35 K309 prohibits full binding to NP.

**Minor Issues**

Reviewer #3: No issues in this section
